# Nuclear reprogramming with a non-integrating human RNA virus

**DOI:** 10.1186/s13287-015-0035-z

**Published:** 2015-03-26

**Authors:** Christopher B Driscoll, Jason M Tonne, Moustafa El Khatib, Roberto Cattaneo, Yasuhiro Ikeda, Patricia Devaux

**Affiliations:** Department of Molecular Medicine, and Virology and Gene Therapy Graduate Track, Mayo Clinic College of Medicine, 200 First Street SW, Rochester, MN 55905 USA

## Abstract

**Introduction:**

Advances in the field of stem cells have led to novel avenues for generating induced pluripotent stem cells (iPSCs) from differentiated somatic cells. iPSCs are typically obtained by the introduction of four factors—OCT4, SOX2, KLF4, and cMYC—via integrating vectors. Here, we report the feasibility of a novel reprogramming process based on vectors derived from the non-integrating vaccine strain of measles virus (MV).

**Methods:**

We produced a one-cycle MV vector by substituting the viral attachment protein gene with the green fluorescent protein (GFP) gene. This vector was further engineered to encode for OCT4 in an additional transcription unit.

**Results:**

After verification of OCT4 expression, we assessed the ability of iPSC reprogramming. The reprogramming vector cocktail with the OCT4-expressing MV vector and SOX2-, KLF4-, and cMYC-expressing lentiviral vectors efficiently transduced human skin fibroblasts and formed iPSC colonies. Reverse transcription-polymerase chain reaction and immunostaining confirmed induction of endogenous pluripotency-associated marker genes, such as SSEA-4, TRA-1-60, and Nanog. Pluripotency of derived clones was confirmed by spontaneous differentiation into three germ layers, teratoma formation, and guided differentiation into beating cardiomyocytes.

**Conclusions:**

MV vectors can induce efficient nuclear reprogramming. Given the excellent safety record of MV vaccines and the translational capabilities recently developed to produce MV-based vectors now used for cancer clinical trials, our MV vector system provides an RNA-based, non-integrating gene transfer platform for nuclear reprogramming that is amenable for immediate clinical translation.

**Electronic supplementary material:**

The online version of this article (doi:10.1186/s13287-015-0035-z) contains supplementary material, which is available to authorized users.

## Introduction

Human pluripotent stem cells may replace non-functional tissues because of their unique capability of giving rise to any cell type in the body. Advances in stem cell research resulted in several processes to generate induced pluripotent stem cells (iPSCs) from adult somatic cells. These iPSCs are typically obtained by the introduction of three to four factors such as OCT4, SOX2, KLF4, and cMYC, which are highly expressed in embryonic stem cells. Derived iPSCs demonstrate properties that are very similar to those of embryonic stem cells [1-3]. Generation of iPSCs from the patient’s own tissues allows novel autologous stem cell therapies while circumventing immunological mismatch and ethical issues associated with the use of embryonic cell sources.

Several vectors have been developed to deliver pluripotency-associated genes or proteins for cellular reprogramming, including integrating lentiviral (LV) and retroviral vectors, RNAs, proteins, and plasmids [4-10]. All of these carriers were used to reprogram fibroblasts into iPSCs. However, efficient reprogramming of patient-derived somatic cells is still challenging. In addition, the use of integrating vectors adds concerns over tumorigenicity because of insertional mutagenesis.

Recently, Sendai virus (SeV), a murine *Paramyxovirus* with a mutant fusion (F) glycoprotein, was developed as an efficient RNA-based gene delivery vector [11,12]. This vector allows a robust and sustained expression of foreign genes. SeV is an enveloped virus with a non-segmented negative-strand RNA genome [13]. Its life cycle/RNA replication occurs in the cytoplasm without DNA intermediates, minimizing the risk of vector genome (RNA) integration into the host genome [14,15]. Efficient, integration-free iPSC derivation from patients with type 1 diabetes was described documenting the successful use of this negative-strand RNA virus for nuclear reprogramming [16,17]. While SeV is in a phase I cancer clinical trial [18], its safety credentials in humans are limited. This may become a critical barrier for rapid translation into future iPSC clinical studies.

Measles virus (MV) is a human *Paramyxovirus*, and its vaccine strain has a well-established safety record in nearly a billion children [19]. Like SeV, MV has a non-segmented negative-strand RNA genome, which is transcribed and replicated in the cytoplasm without production of DNA intermediates [13,20-22]. The MV genome contains six genes—N, P, M, F, H, and L—that encode eight proteins: the nucleoprotein (N), the phosphoprotein (P), the matrix protein (M), the two glycoproteins hemagglutinin (H) and fusion protein (F), the viral polymerase (L), and the two accessory proteins V and C [13].

Using reverse genetics, we have generated genomic cDNAs with the coding capacity identical to that of an MV Moraten/Schwartz vaccine strain. These genomic cDNAs have regularly spaced unique restriction sites to assist in mutagenesis and additional transcription unit (ATU) inserted in different positions, allowing expression of foreign genes [23-25]. As MV transcription is gradually reduced the farther the polymerase travels down the genome, the different location of these ATUs along the genome allows differential levels of expression of the transgene. These vectors, which have been used to express antigens from different pathogens [23,26-32], can accommodate up to 6 kilobases of foreign genetic material, allowing insertion of several genes. MV was also developed as an oncolytic vector [33,34]. Four phase I or II clinical trials are currently active, and no serious adverse events have been reported [33,35,36].

We present here the first application of MV as a vector for nuclear reprogramming. We have produced a one-cycle MV vector expressing one of the four reprogramming factors: OCT4. We conducted a proof-of-principle experiment by substituting LV expressing OCT4 with an MV-based vector expressing OCT4 in a four-vector reprogramming system. We reprogrammed newborn somatic cells into iPSCs and characterized these MV-derived iPSCs for expression of stem cell markers, induction of endogenous pluripotency-associated genes, and pluripotency.

## Methods

All animal studies were approved by the Mayo Institutional Animal Care and Use Committee. The human cell line used in this study was purchased from the American Type Culture Collection (Manassas, VA, USA) and did not require consent or the need for Mayo Institutional Review Board approval.

### Cells and viruses

Vero-H2 cells and the helper 293-3-46-H2 cell line were produced by transducing Vero cells (ATCC #CCL81; American Type Culture Collection) and the helper 293-3-46 cell line [37] with an LV encoding for the MV hemagglutinin gene. Vero-H2, Vero, and 293T cells were maintained in Dulbecco’s modified Eagle’s medium (DMEM) (Mediatech Inc., Herndon, VA, USA) supplemented with 10% fetal calf serum (FCS), 1% penicillin/streptomycin (Mediatech) (DMEM-10), and helper 293-3-46-H2 cells in DMEM-10 containing 1.2 mg/mL G418 (Mediatech). The human BJ cells, neonatal foreskin human cells, were purchased from the American Type Culture Collection (ATCC #CRL 2522). BJ cells were maintained in DMEM-10 containing 0.1 mM of non-essential amino acids. iPSCs were maintained in 80% Pluriton (Stemgent, Cambridge, MA, USA) and 20% mTeSR1 (Stemcell Technology, Vancouver, BC, Canada) mixed medium supplemented with 1% penicillin/streptomycin.

Recombinant MVs were generated as described by Radecke *et al*. [37]. Briefly, the helper 293-3-46-H2 cell line was transfected by calcium phosphate precipitation by using a ProFection kit (Promega, Madison, WI, USA) with two plasmids, one coding for the relevant MV genome and the other for the MV polymerase (pEMCLa). Three days after transfection, the helper cells were overlaid on Vero-H2 cells, the appearance of infectious centers was monitored, and single viruses were propagated on Vero-H2 cells. To prepare virus stocks, Vero-H2 cells were infected at a multiplicity of infection (MOI) of 0.03 and incubated at 32°C for 4 to 5 days or until 95% of the cells expressed green fluorescent protein (GFP). Cells were scraped in Opti-MEM (Life Technologies, Grand Island, NY, USA), and particles were released by two freeze-thaw cycles. Virus stock titers were determined by 50% end-point dilution (tissue culture infectious dose 50, or TCID_50_) on Vero-H2 cells by using the Spearman-Kärber method [38].

### Expression plasmids, full-length measles virus infectious cDNA, and lentiviral vector vectors

To produce the p(+)MVvac2(OCT4)P full-length cDNA vector, the open reading frame of OCT4 was inserted into the ATU located between P and M genes in the full-length cDNA of pB(+)MVvac2(ATU) [23,24]. Cloning was performed by using the following primers: 5′-acgcgtcgtacgtcgcgaatgtacaacatgatggagac-3′ and 5′-gcgcgctacgtatcgcgatcacatgtgtgagaggg-3′ and the In-Fusion HD Cloning kit (Promega). To generate one-cycle virus, the H gene was substituted with the GFP gene. The GFP open reading frame was amplified by using the following primers: 5′-cggtagttaattaaaacttagggtgcaagatcatcgatatcgcgaatggtgagcaagggcg-3′ and 5′-gacgagctgtacaagtagttcgcgacccgggaagatggaaccaat-3′. The substitution was performed in the pCG-Hvac2 plasmid by using the *PacI* and *SmaI* sites. To obtain the full-length p(+)MVvac2(OCT4)P-ΔH(GFP), cDNA was then obtained by transfer of a *PacI-SpeI* fragment in the p(+)MVvac2 [24]. The LV encoding the MV H (LV-H) was produced by cloning the open reading frame of H into the pSIN LV by using the *MluI* and *NotI* restriction sites.

LV particles generated from the plasmids pSIN-H, pSIN-GFP, pSIN-OCT4, pSIN-SOX2, pSIN-KLF4, and pSIN-cMYC were produced as described previously [39]. Briefly, the cDNA vectors were co-transfected into 293 T cells along with a packaging plasmid (pCMVR8.91) and a plasmid encoding VSV-G (vesicular stomatitis virus G protein) for pseudotyping (pMD-G) [40] by using the Fugene 6 kit (Life Technologies) in accordance with the instructions of the manufacturer. Two days after transfection, the supernatant was centrifuged at 3,000 revolutions per minute for 15 minutes, filtered on 0.45-μm filters, and aliquoted and stored at −80°C. An LV-GFP stock was always produced in parallel with any LV productions and used for semi-quantitative viral titration. LV titration was performed by transduction of BJ cells by using 25, 50, 75, 100, 150, and 200 μL of viral stock. The lowest volume (on average, 50 μL) of viral stock determined to give 90% to 100% of green (GFP) fluorescent cells was then used in future transduction experiments. The Mayo Clinic Institutional Biosafety Committee has approved all vectors, viruses, and experiments performed in this study.

### H surface expression and infection efficiency quantification by flow cytometry

Vero, Vero-H2, 293-3-46, and 293-3-46-H2 cells were incubated with rabbit anti-H diluted in phosphate-buffered saline (PBS)-4% FCS [41] for 1 hour at 4°C. After two washes, the cells were incubated with a fluorescein isothiocyanate (FITC)-labeled anti-rabbit secondary antibody diluted in PBS-4% FCS (Jackson ImmunoResearch Laboratories, West Grove, PA, USA) for 1 hour at 4°C. The cells were washed twice and fixed in PBS-2% paraformaldehyde (PFA) (Affymetrix, Inc., Cleveland, OH, USA). Flow cytometry data were acquired on a Becton Dickinson FACS Calibur (Becton Dickinson, Franklin Lakes, NJ, USA) and analyzed by using Flowjo software.

BJ cells were transduced or not with MV(OCT4) vector at an MOI of 6 for 48 hours. The cells were washed once with PBS and detached with Versene. After two washes with PBS, the cells were fixed with PBS-2% PFA. GFP-expressing cells were analyzed by flow cytometry as described above.

### Measles virus vector infectivity

Vero and Vero-H2 cells were infected with MV(OCT4) or MV control virus at an MOI of 0.05 or 0.1 for 2 hours at 37°C in Opti-MEM. After 2 hours, the inoculum was removed, cells were washed once, and 1 mL of medium was added to the cells. Cells were then incubated at 37°C. Cells were collected at two different time points: 24 and 48 hours post-infection. Cells were collected in the 1 mL of supernatant and freeze-thawed once to release cell-associated virus. Viral titers were determined by 50% end-point dilution (tissue culture infectious dose 50, TCID_50_) on Vero-H2 cells by using the Spearman-Kärber method [38].

### Reprogramming of human foreskin fibroblasts

Reprogramming of BJ foreskin cells (5 × 10^4^) was performed by transduction with three LVs expressing SOX2, KLF4, or cMYC and one MV vector expressing OCT4. Control reprogramming was performed by using either four LVs or three LVs only (without OCT4). The amount of LV required for the most efficient reprogramming was tested by transducing the cells with different amounts of the four LVs, starting with the lowest dose of virus determined to give 90% to 100% of GFP-positive cells. The amount of LV showing the highest efficiency to produce iPSC colonies was used to reprogram with the MV(OCT4) vector. Six days after transduction, cells were detached with Versene and replated on Matrigel-coated plates (BD Biosciences, San Jose, CA, USA) and fed with the serum-free human iPSC media consisting of Pluriton medium (Stemgent) supplemented with 20% (vol/vol) mTeSR-1 maintenance media (Stemcell Technologies). One to three weeks after vector infection, reprogrammed cells began to form iPSC-like colonies, and at 3 to 6 weeks, colonies were picked on the basis of size and morphology.

### Immunoblot analysis of cell extracts

BJ and Vero cells (5 × 10^4^) were transduced with LV(OCT4) or MV(OCT4) vectors by using the same conditions used for the reprogramming transduction. After 36 hours, the cells were processed as described elsewhere [24]. After fractionation on 4% to 15% SDS-polyacrylamide gels (Bio-Rad Laboratories, Hercules, CA, USA) and transfer to polyvinylidene difluoride membranes (Immobilon-P; Millipore, Billerica, MA, USA), the samples were subjected to enhanced chemiluminescence detection by using the antibodies indicated. The following antibodies were used: rabbit anti-OCT4 (Cell Signaling Technology, Inc., Danvers, MA, USA) and anti-β-actin (Sigma-Aldrich, St. Louis, MO, USA) as a loading control.

### Immunostaining and confocal microscopy

Cells (2.5 × 10^4^ Vero or BJ) were plated in chamber slides (Lab Tek II Chamber Slide system; Nalge Nunc International Corp., Naperville, IL, USA) and were transduced with either the LV(OCT4) or MV(OCT4) by using the same conditions used for the reprogramming transduction. Thirty hours after transduction, cells were washed once with PBS and fixed with PBS-4% PFA for 15 minutes. Cells were then permeabilized with PBS-4% PFA-0.1% Triton X-100 for 15 minutes, washed with PBS, incubated for 1 hour in blocking solution (PBS-4% FCS), and immunostained by using a rabbit antibody directed against the human OCT4 (#2750; Cell Signaling Technology). Incubations were performed overnight at 4°C in PBS-2% FCS. After five washes, the cells were incubated for 1 hour with a Rhodamine anti-Rabbit secondary antibody (Jackson ImmunoResearch Laboratories). After the five washes, cells were mounted with Prolong Gold Antifade reagent containing DAPI (4′,6-diamidino-2-phenylindole) (Life Technologies) and analyzed with a Zeiss LSM 780 confocal microscope (Zeiss, Oberkochen, Germany). Images were analyzed by using the Zen black software from Zeiss.

For the characterization of undifferentiated iPSC clones, iPSCs were plated in Matrigel-coated chamber slides, fixed, and permeabilized as described above. After blocking, the cells were incubated overnight with the following antibodies: anti-MV-N (Cl25 [42]), SSEA-1, SSEA-4, TRA-1-60, TRA-1-81 (#SCR001; Millipore), OCT4 (#2750; Cell Signaling Technology), SOX2 (#2748; Cell Signaling Technology), and NANOG (#ab21624; Abcam, Cambridge, MA, USA). After three washes, secondary antibodies AlexaFluor 488 anti-mouse and AlexaFluor 488 anti-rabbit (Life Technologies) were incubated for 1 hour at room temperature. After three washes, the cells were mounted and analyzed as described above. Finally, the alkaline phosphate staining was performed with an Alkaline Phosphatase Detection Kit (#SCR001; Millipore).

### Cellular and viral gene transcription

Total RNA from one well of a six-well plate of iPSCs was isolated with the Trizol reagent (Life Technologies) and used for semi-quantitative RT-PCR analysis. Reverse transcription was performed by using the RNA to cDNA EcoDry TM Premix (Oligo dT) kit (Clontech, Mountain View, CA, USA). Platinum Taq DNA polymerase (Life Technologies) was used for PCRs. Reactions were performed in accordance with the manufacturer protocols. Primers used to measure transcription levels of pluripotency markers (OCT4, SOX2, KLF4, NANOG, GDF3, hTERT, and cMYC) were previously described [17]. Primers used to measure transcription levels of the N mRNA in iPSC clones were 5′-cataaggctgttagagg-3′ and 5′-cccagaatcatgttgaat-3′.

### Flow cytometry of pluripotency markers

MV- and LV-iPSC-derived cells were dissociated into single cells by using Versene. Extracellular staining was performed by incubating the cells (2 × 10^5^) with the primary antibody TRA-1-60, TRA-1-81, SSEA-1, and SSEA-4 for 1 hour on ice in PBS supplemented with 2% FCS and 0.05% NaN3. After two washes with PBS with 2% FCS and 0.05% NaN3, the cells were incubated with an FITC-labelled anti-mouse secondary antibody (Jackson Research Laboratories) for 1 hour. Intracellular OCT4 antibody staining was performed by fixing 4 × 10^5^ cells with PBS-2% PFA for 20 minutes. The cells were washed with PBS-2% FCS-0.05% NaN3 once and then permeablised for 20 minutes with 500 μL of saponin permeabilization buffer (SPB) (PBS supplemented with 1 mg/mL Saponin and 1% bovine serum albumin) at room temperature. The primary and secondary antibodies were incubated for 1 hour each in the SPB on ice. Finally, the cells were fixed in 2% PFA. Flow cytometry data were acquired on a Becton Dickinson FACS Calibur (Becton Dickinson) and analyzed by using Flowjo software.

### Cytogenetic analysis

Conventional cytogenetic analysis was performed on 20 metaphase cells of the iPSC clones. Chromosomes were banded in accordance with standard methods for high-resolution G-banding. Cells were captured and karyotyped by using a CytoVision Karyotyping System (Genetix, New Milton, UK).

### Spontaneous differentiation assay

For spontaneous differentiation, MV- and LV-derived iPSC clones were dissociated by using Versene and cultured on low-adhesion plates in iPSC medium for embryoid body (EB) formation. EBs were cultured in suspension for 10 days and then allowed to adhere to a Matrigel-coated plate in DMEM with 20% fetal bovine serum and further cultured for 10 to 14 days. Differentiated cells were analyzed for markers of three germ layers. FOXA2 for endoderm (#07-633; Millipore), beta III tubulin (#ab41489; Abcam) for ectoderm, and CD31 (#sc-1506; Santa Cruz Biotechnology, Dallas, TX, USA) for mesoderm were used to stain the EB-derived cells.

### Teratoma formation and *in vivo* differentiation of induced pluripotent stem cells

All studies were approved by the Mayo Clinic Institutional Animal Care and Use Committee. Female severe combined immunodeficiency (SCID)-beige mice (Charles River Laboratories, Wilmington, MA, USA) were anesthetized, and the kidney was exposed for iPSC transplantation under the kidney capsule. To this end, a small incision was made in the kidney capsule, and a blunt needle was used to create a pocket under the kidney capsule. After iPSC injection, the kidney was placed back into the abdomen, and the incision was closed. Mice were maintained for 4 weeks and sacrificed before harvesting of iPSC-transplanted kidneys. Tissues were embedded and flash-frozen in O.C.T Compound (Sakura Finetek USA, Inc., Torrance, CA, USA). Cryosections (7-μm) were obtained and immediately fixed in 4% PFA for 30 minutes at room temperature before proceeding to hematoxylin-and-eosin staining.

### Differentiation of induced pluripotent stem cells into cardiomyocyte-like cells

iPSCs were plated in Matrigel-coated chamber slides. When the cells reached confluency, cardiac differentiation was started by using a PSdif-Cardio cardiomyocyte differentiation kit (StemRD, Burlingame, CA, USA). After 10 days of culture, the first contracting cells were observed under light microscopy. Video of the contracting cardiomyocyte-like cells was obtained by using a Zeiss Axiovert 200 M microscope and the ApoTome imaging system. The software used for the movie analysis is Axiovision from Zeiss. Immunostaining for α-actinin (#A7811; Sigma-Aldrich), a marker of microfilaments, and troponin (#Ab8295; Abcam) marker specific for the cardiac muscle was analyzed with a Zeiss LSM 780 confocal microscope.

## Results

### Production of a ‘one cycle’ measles virus vector expressing the reprogramming factor OCT4

**Figure 1:**
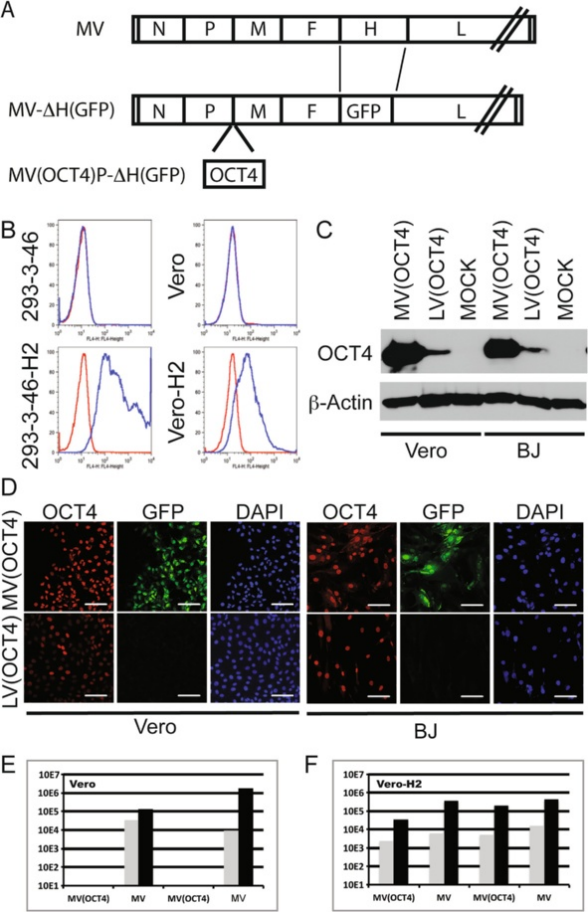
**Generation and characterization of a one-cycle measles virus (MV) vector expressing OCT4. (A)** Top: MV genome. Middle: Genome of MV expressing green fluorescent protein (GFP) but no H protein. Bottom: Genome of MV expressing OCT4 and GFP but no H protein. The MV antigenome (plus strand) is represented with its 5′ end on the left; the six genes are indicated by capital letters. **(B)** Flow cytometry analysis of H expression on 293-3-46 (left) and Vero (right) cells after LV-H transduction. Top graphs, mock-transduced cells. Bottom graphs, LV-H transduced cells. **(C)** Immunoblot analysis of OCT4 expression. BJ and Vero cells were infected with the indicated vector and after 36-hour cell extract were analyzed by SDS-PAGE. OCT4 antibody against OCT4 protein was used. Uninfected BJ and Vero cells (Mock) were used as controls. β-actin antibody was used as control of protein load. **(D)** Immunofluorescence analysis of OCT4 expression. Vero and BJ cells were infected with the indicated vector for 36 hours and analyzed by immunostaining and confocal microscopy. The cells were fixed, permeabilized, and stained with antibody to OCT4 (red). Nuclei were counterstained by 4′,6-diamidino-2-phenylindole (DAPI) (blue). GFP (green) was expressed during infection. Scale bars: 100 μm. **(E)** Titers of cell-associated and released virus produced upon infection of Vero cells with MV(OCT4) or MV, determined at 24 hours (gray columns) or 48 hours (black columns) post-infection. **(F)** Titers of cell-associated and released virus produced upon infection of Vero-H2 cells with MV(OCT4) or MV, determined at 24 hours (gray columns) or 48 hours (black columns) post-infection.

We had previously generated p(+)MVvac2(ATU)P, a full-length cDNA clone with equivalent coding capacity as the US Food and Drug Administration-approved Moraten vaccine strain encoding for an ATU between the P and M genes [23]. From this location, genes are transcribed at an intermediate level, allowing position-dependent increase or decrease of protein expression levels if needed. The OCT4 open reading frame was inserted in the ATU resulting in p(+)MVvac2(OCT4)P. To produce a recombinant ‘one cycle’ MV genome and to avoid cell fusion during reprogramming, we substituted the MV attachment protein H gene with GFP. Together with the F protein, the H protein forms the protein complex that fuses membranes and allows virus entry [43]. In the absence of H, membrane fusion cannot occur and infection does not spread [43]. To produce such ‘one cycle’ virus, the GFP gene was substituted for the H gene of the genomic plasmid p(+)MVvac2(OCT4)P (the viral genome in this plasmid is shown in Figure 1A, top line). To allow virus particle production, we generated a rescue cell line expressing the MV H protein, which was named 293-3-46-H2, and a propagation cell line that we named Vero-H2. These cell lines were transduced with an LV expressing MV H, and H cell surface expression was confirmed by immunostaining and flow cytometry analyses (Figure 1B, lower panels, blue histograms). The ‘one cycle’ MV-vector was named MV(OCT4)P-ΔH(GFP) (Figure 1A, bottom) and abbreviated as MV(OCT4).

We analyzed the expression of OCT4 by immunoblot and immunofluorescence and compared it with the OCT4 expression after LV transduction (Figure 1C, D). After infection with MV(OCT4), both Vero and BJ cells showed a stronger expression of OCT4 compared with the control cells transduced with the LV(OCT4) (Figure 1C, compare first and second lines in each cell line). Although the higher infection efficiency of MV(OCT4) in Vero cells could partly account for the higher amount of OCT4 expression (Figure 1D, Vero, compare red panels, left half), in BJ cells infection levels were equivalent (Figure 1D, compare red panels, right half). Only single infected cells were detected, indicating that our vector does not fuse cells that do not express MV-H, as expected (Figure 1D, center panels, top row). To confirm the production of a ‘one cycle’ MV vector, we performed a one-step infection on cells expressing (Vero-H2) or not expressing (Vero) the MV-H protein. Cells were infected at an MOI of 0.05 or 0.1, and viral titers were determined 24 or 48 hours after infection. Figure 1E presents the viral titers of Vero infected with MV(OCT4) and control MV. Whereas a high viral titer is observed with the control MV, no virus was produced with the MV(OCT4). In contrast, the expression of MV-H at the cell surface restores efficient virus production (Figure 1F). These results confirmed that MV(OCT4) is a ‘one cycle’ vector and that expression of MV-H in trans is required for viral spread.

### Reprogramming using MV(OCT4) instead of LV(OCT4) leads to production of induced pluripotent stem cell-like colonies

**Figure 2:**
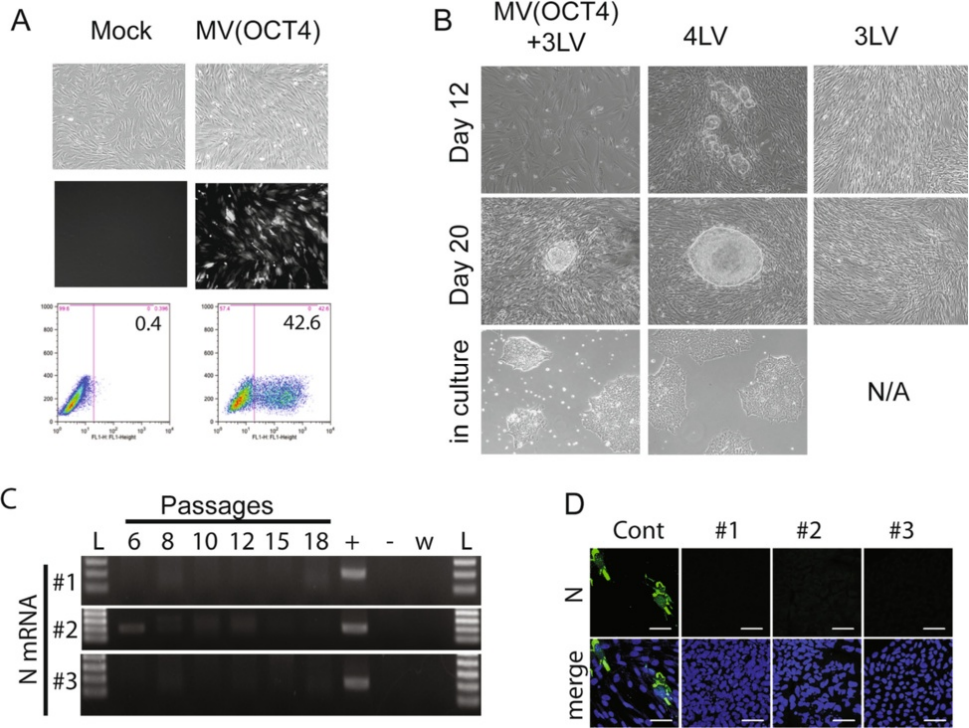
**Generation of induced pluripotent stem cell (iPSC)-like clones by MV(OCT4) in combination with three lentiviral vectors (LVs). (A)** Level of BJ cells transduction with MV(OCT4). BJ cells were infected with MV(OCT4) and three LVs encoding SOX2, KLF4, and cMYC (left panel). Forty-eight hours after infection, pictures were taken under phase contrast (top panels) or fluorescence (middle panels). Cells were then collected, and the number of cells expressing GFP was quantified by flow cytometry (bottom panels). **(B)** Reprogramming of BJ cells. BJ cells were infected with MV(OCT4) and three LVs encoding SOX2, KLF4, and cMYC (left panels); four LVs encoding OCT4, SOX2, KLF4, and cMYC (middle panels); or three LVs encoding SOX2, KLF4, and cMYC (right panels). Cells were observed under light microscopy, and pictures of iPSC-like clones were taken at different time points, as indicated. Third row panels show iPSC clones in culture. **(C)** Loss of viral gene expression after passaging. Nucleoprotein (N) mRNA expression levels were analyzed in iPSC clones by reverse transcription-polymerase chain reaction at passages 6, 8, 10, 12, 15, and 18. Control (Cont): (+) BJ cells infected with MV(OCT4), (−) BJ mock infected, (w) water, (L) ladder. **(D)** Immunofluorescence analysis of N protein expression. iPSC clones were analyzed by immunostaining and confocal microscopy. The cells were fixed, permeabilized, and stained with antibody to N (fluorescein isothiocyanate, green). Nuclei were counterstained by 4′,6-diamidino-2-phenylindole (DAPI) (blue). Scale bars: 50 μm. MV, measles virus.

To assess whether MV vectors can be used for nuclear reprogramming, we substituted MV(OCT4) for LV(OCT4) for the reprogramming of human foreskin BJ fibroblast cells into iPSCs. At day 0, the cells were transduced overnight with three LVs (50 μL each) encoding for SOX2, KLF4, and cMYC and infected with MV(OCT4) at an MOI of 6. Forty-eight hours after infection, an average of 40% to 45% of the cells expressed GFP, indicating efficient transduction (Figure 2A). Media were changed every other day. When the cells became too confluent, at day 6, they were split and seeded into two Matrigel-coated wells of a six-well plate. MV transcription and replication occur only in dividing cells, and thus it is important to keep the cells in an under-confluent stage to allow continuous expression of the reprogramming factors until iPSC clones appear. Although in the first week we noticed some toxicity of the MV vector, GFP-expressing cells remained visible 21 days after transduction, indicating long-lasting expression of the vector in the cells. While sharp-edge, flat, and tightly packed iPSC-like colonies appeared within 2 weeks for 4LV control reprogramming (Figure 2B, 4LV panel, day 12), it took around 20 days for the first iPSC-like colonies to appear for the MV(OCT4) + 3LV (Figure 2B, compare left and control columns). This suggests that the process of reprogramming takes longer with the MV vector. When the clones were large enough, around 30 to 35 days post-transduction for the MV-derived iPSC clones, they were picked and propagated individually on Matrigel-coated plates, first into a 96-well plate (passage P1), then into a 24-well plate (P2), a 12-well plate (P3), and finally six-well plates (P4 and higher) for conservation and further analysis. From the day of picking, the clones are passaged every 4 days; thus, at passage 6, the time of our first analysis, the clones were at around 54 to 59 days post-transduction. Three clones from MV(OCT4) + 3LV infection (#1, #2, and #3) and one clone from the control 4LV transduction were further analyzed. Recovery of iPSC-like clones was successful numerous times in the same conditions. Different individuals obtained similar yields from two to 10 expandable clones per 5 × 10^4^ transduced BJ cells. In contrast, the yield obtained with the 4LV was higher, between 40 to 50 expandable clones per 5 × 10^4^ transduced BJ cells. Thus, the yield of reprogramming with one MV + 3LV is lower than with the 4LV. We performed a negative control reprogramming with only the three LVs encoding SOX2, KLF4, and cMYC and were unable to detect iPSC clone formation (Figure 2B, right panels), confirming that the presence of OCT4 is required for iPSC formation.

To measure the longevity of MV vector expression in the iPSC-like clones, we performed reverse transcription-polymerase chain reaction (RT-PCR) to detect the mRNA from the nucleoprotein (N) gene, which is the highest expressed gene during MV transcription. The three clones were propagated for over 18 passages and RNA was collected at passages 6, 8, 10, 12, 15, and 18 (Figure 2C). Only clone #2 showed some residual expression at passages 6 and 8 (53 to 59 and 61 to 67 days post-transduction, respectively), suggesting that the MV vector is eventually eliminated from the cells. Immunofluorescence analysis confirmed the absence of N expression at passage 6 in all three clones (Figure 2D).

### Measles virus-derived induced pluripotent stem cell-like clones express pluripotency markers

**Figure 3:**
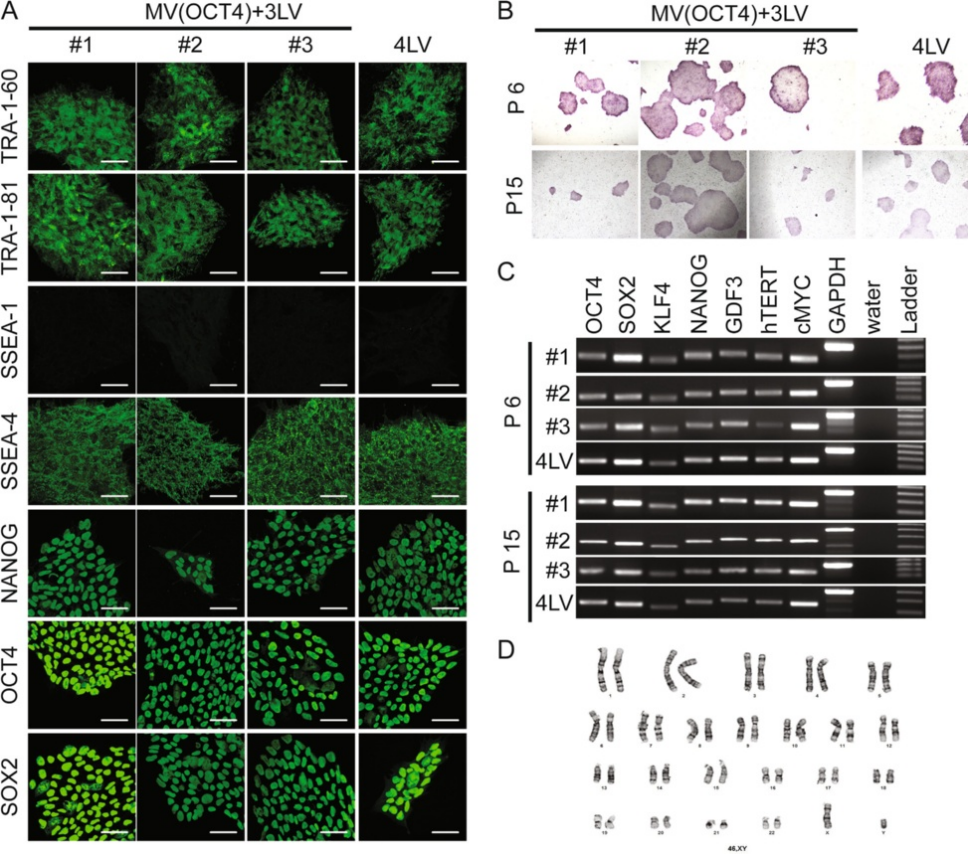
**Expression of pluripotency-associated markers in measles virus (MV)-derived induced pluripotent stem cell (iPSC)-like clones.** Three MV- and one lentiviral vector (LV)-derived iPSC clone (#1, #2, #3, and 4LV) were cultured under feeder-free conditions on a Matrigel-based slide and examined for expression of human pluripotent stem cell markers by immunofluorescence **(A)** or alkaline phosphatase **(B)**. Both passages 6 and 15 were tested. Scale bars: 50 μm. **(C)** Reverse transcription-polymerase chain reaction analysis assessing transcription of key pluripotency-associated markers (OCT4, SOX2, KLF4, NANOG, GDF3, hTERT, and cMYC) using total cellular RNA of the same four iPSC clones at passages 6 and 15. GAPDH (glyceraldehyde 3-phosphate dehydrogenase) is the cellular internal control, and water is the negative control. **(D)** G-banding chromosome analysis of iPSC clone #3.

To further characterize the clones produced with the MV(OCT4) + 3LV, we tested these cells for expression of several markers of pluripotency. All clones expressed human pluripotency-associated markers SSEA-4, TRA-1-60, TRA-1-81, OCT4, SOX2, and NANOG at passage 6 (Figure 3A). Similar analyses were performed at passage 15, and all clones remained positive for these markers (Additional file 1: Figure S1). All tested iPSC-like clones were negative for SSEA-1 expression at both passages 6 and 15 (Figure 3A and Additional file 1: Figure S1). The three MV-derived clones were positive for alkaline phosphatase activity at both passages 6 and 15 (Figure 3B). We also performed semi-quantitative RT-PCR analysis to confirm the induction of endogenous pluripotency-associated markers including OCT4, SOX2, KLF4, NANOG, GDF3, hTERT, and cMYC. All markers were expressed in the three clones tested in a similar way as in the 4LV clone control (Figure 3C). The markers of pluripotency remained unchanged from passages 6 to 15 (Figure 3B, C), suggesting the maintenance of an undifferentiated state of the iPSC clones produced by the MV(OCT4) vector. Finally, we performed flow cytometry analysis for some of the markers (TRA-1-60, TRA-1-81, SSEA-4, and OCT4) at passages 6 and 16. Expression levels of marker remained the same after 10 additional passages, as did the number of cells expressing the markers, confirming the stability of the clones (Additional file 2: Figure S2). All MV-derived-iPSC clones showed identical marker profiles and had similar characteristics to the clones derived with 4LV, suggesting that they all have reprogrammed into iPSCs.

Karyotyping analysis of derived iPSC clones was performed at passage 17. Most of the cells from clones #1 and #3 have normal diploid karyotypes (Figure 3D and Additional file 3: Figure S3). Only a small number of cells showed an abnormal karyotype with trisomy 12 or 14 (Additional file 3: Figure S3). Since frequent trisomy has been noted in human iPSCs and embryonic stem cells after prolonged passages [44-46], we conclude that the observed chromosomal abnormalities in these clones are due to continuous *in vitro* passage. Clone #2 showed abnormalities of 20q chromosome and such abnormalities have also been observed in prolonged culture of iPSCs [44-47]. Clone #2 also showed a loss of the Y chromosome. We speculate that this chromosome deficiency was present in the pre-reprogrammed parental fibroblast cell and was not induced during or after the reprogramming process (Additional file 3: Figure S3).

### Measles virus-derived induced pluripotent stem cell clones can differentiate into the three germ lineages or into cardiomyocyte-like cells

**Figure 4:**
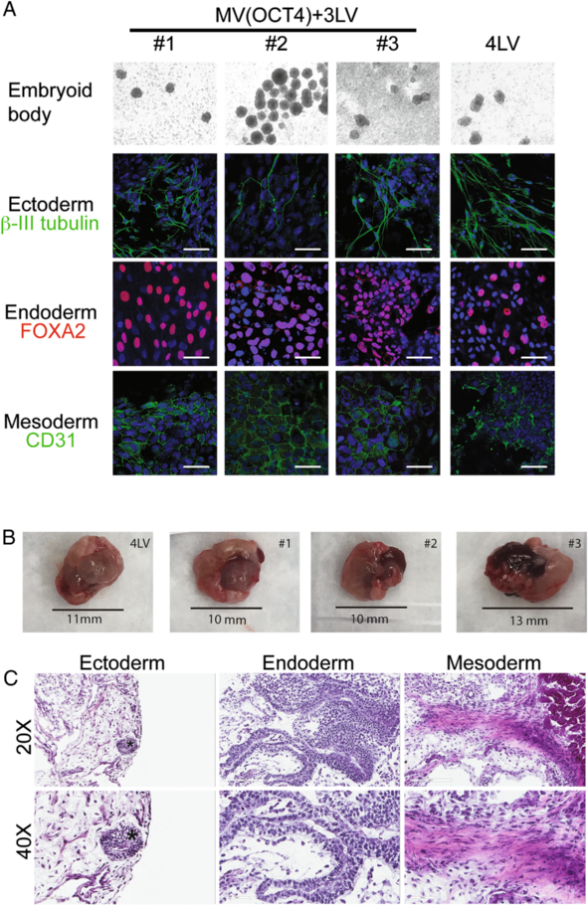
**Spontaneous differentiation of the measles virus (MV)-derived induced pluripotent stem cell (iPSC)-like clones and teratoma formation. (A)** Three MV- and one lentiviral vector (LV)-derived iPSC clone (#1, #2, #3, and 4LV) were analyzed by immunofluorescence for lineage markers for three germ layers (endoderm, mesoderm, and ectoderm). iPSC clones were spontaneously differentiated through embryoid body formation (top row). Pluripotency of derived iPSC clones was verified by generation of cells of ectoderm (β-III tubulin, green, second row), endoderm (FOXA2, red, third row), and mesoderm (CD31, green, bottom row) upon spontaneous differentiation. Nuclei were counterstained by 4′,6-diamidino-2-phenylindole (DAPI) (blue). Scale bars: 50 μm. **(B)** Transplant of iPSC clones into the kidney capsule of severe combined immunodeficiency (SCID)-beige mice resulted in teratoma formation. Pictures of harvested kidneys with iPSC transplant are shown for all clones. **(C)** Representative hematoxylin-and-eosin staining demonstrated multiple lineages within the complex architecture of the tumor, including cells of ectoderm (neuronal rosette-like structures, indicated by an asterisk), endoderm (gut tube-like structures, intestinal epithelial cells), and mesoderm (muscle-like tissue). Magnifications: 20× (top) and 40× (lower).

To confirm that the MV-derived iPSC-like clones are pluripotent, we assessed their differentiation propensity into the three germ layers: endoderm, ectoderm, and mesoderm. To assess their ability to form EBs *in vitro*, iPSCs were cultured in suspension. Indeed, all three clones formed EBs (Figure 4A, top row). After 10 days in culture, the cells were allowed to adhere on Matrigel-coated chamber slides and cultured for an additional 10 days in the presence of 20% FCS to induce their spontaneous differentiation into the three germ lineages. Immunostaining of EB-derived adherent cells detected cells prototypic of the ectoderm (β-III tubulin), endoderm (FOXA2), or mesoderm (CD31) (Figure 4A, second, third, and fourth rows, respectively), documenting multi-lineage propensity of MV-derived iPSCs. Moreover, *in vivo*, iPSCs transplanted under the kidney capsule of SCID-beige mice at a dose of 2 million cells gave rise to 10- to 13-mm outgrowth within 4 weeks (Figure 4B). Tissue histology revealed iPSC differentiation into ectoderm lineages denoted by neuronal rosette-like structures (Figure 4C, left, indicated with an asterisk), endoderm lineages composed of gut tube-like structures, intestinal epithelial cells (Figure 4C, center), and mesoderm lineages indicated by muscle-like tissue (Figure 4C, right). These data document the multi-lineage propensity of MV-derived iPSCs.

**Figure 5:**
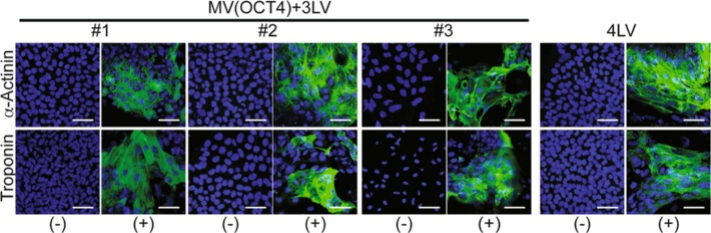
**Cardiac differentiation of measles virus (MV)-derived induced pluripotent stem cell (iPSC)-like clones.** Three MV- and one lentiviral vector (LV)-derived iPSC clone (#1, #2, #3, and 4LV) were analyzed for cardiac differentiation. Immunofluorescence for cardiac markers is shown. iPSCs before (−) and after (+) cardiac differentiation were fixed, permeabilized, and stained with antibody to α-actinin (green) or troponin (green). Nuclei were counterstained by 4′,6-diamidino-2-phenylindole (DAPI) (blue). Scale bars: 50 μm.

Next, we used a step-wise differentiation protocol to test the proficiency of MV-derived iPSCs to generate cardiac progenitor cells *in vitro*. Ten days into differentiation into the mesodermal pathway, contracting cells were observed (Additional file 4: Video V1 shows LV-differentiated cardiomyocyte-like cells, and Additional file 5: V2 shows MV(OCT4)-derived differentiated cardiomyocyte-like cells). Thus, iPSCs differentiated into cardiomyocyte-like cells. To assess whether the contracting cells express a specific marker of cardiac differentiation such as α-actinin and troponin, we performed immunostaining. Although these two markers were not expressed on the iPSCs before differentiation (Figure 5, (−)), both were expressed at a high level in the differentiated cells (Figure 5, (+)). Thus, MV-derived iPSCs can differentiate into contracting cardiomyocyte-like cells.

## Discussion

In this study, we established the feasibility of using a non-integrating, non-pathogenic human negative-strand RNA virus as a vector to express factors required for the reprogramming of human cells into iPSCs. We substituted LV-based vector expressing OCT4 with an MV-based vector expressing this factor and produced iPSC clones. These clones have the same characteristics as those obtained with 4 LVs. Our results indicate that MV replication did not affect the process of reprogramming and that OCT4 was produced in sufficient quantity. Thus, MV can be used as a vector for efficient nuclear reprogramming.

### Negative-strand RNA viruses as reprogramming tools

Among the main advantages of non-integrating negative-strand RNA viruses over other systems are that the entire viral cycle occurs in the cytoplasm and that there are no DNA intermediates [13]. No DNA integration events were ever reported for the RNA genome of these viruses because of these two characteristics. Thus, there is no insertional mutagenesis risk into the genome of the iPSCs. It is possible to insert large genes in the MV genome because particle size is not constrained by icosahedral symmetry [30]. Here, we showed for the first time that the vaccine strain of the human negative-strand RNA virus, with established safety records in humans, can provide a unique and clinically applicable reprogramming platform.

The balance between the levels of expression of reprogramming factors is important for efficient reprogramming [12,48-50]. For example, Papapetrou *et al*. have demonstrated that optimal reprogramming efficiency depends on accurate OCT4 dosage and that the relative expression levels of reprogramming factors can affect the efficiency of human iPSC derivation [49]. We found that the levels of OCT4 expression in MV-infected BJ cells was at least 10 times higher than those observed in cells infected with LV(OCT4). We also noticed that the reprograming efficiency with MV + 3LV was lower than with the 4LV. This may be due to the overexpression of OCT4, and further fine-tuning of the expression levels of the four reprogramming factors would improve the reprogramming efficiency. Negative-strand RNA virus transcription is gradually reduced at each gene junction, allowing expression of foreign genes at different levels. Indeed, genomic location was exploited to optimize the expression levels of the KLF4 factor in a Sendai vector [12].

We have attempted to reprogram BJ cells with an MV expressing SOX2, along with LV encoding OCT4, KLF4, and cMYC. Some of the transduced cells formed neural stem cell-like colonies but not iPSC colonies. SOX2 protein expression analysis indicated substantially higher levels of SOX2 expression from the MV vector when compared with LV(SOX2) (unpublished data). At present, we are further analyzing these clones for potential characteristics of neural stem cells, as SOX2 overexpression can lead to a direct reprogramming into neural stem cells [51]. Reduced SOX2 expression through moving its gene to a downstream location in the MV genome [52] would improve the iPSC reprogramming.

### Measles virus versus Sendai virus vectors

SeV vectors are based on a temperature-sensitive strain, which requires an increase in temperature for a more efficient elimination of the viral genome after reprogramming into iPSCs [11,16]. Without this temperature control, expression of the N protein was monitored in some iPSCs after 30 to 40 passages (Y. Ikeda, unpublished data). Here, we were unable to detect N transcripts after 6 to 8 passages, indicating that viral clearance likely occurs rapidly and that MV vectors might not require an intervention to accelerate elimination. If genome elimination were an issue, we could insert a microRNA target sequence in the MV genome to accelerate virus elimination [53,54]. Another advantage of our new MV vector is that it is deficient in its main immunogenic determinant, the H protein.

Finally, the clinical usage of SeV is limited, including a phase I clinical trial with a SeV vector expressing the human fibroblast growth factor-2 (FGF-2) gene to treat peripheral arterial disease [18]. In contrast, MV has a proven record for safety as a pediatric vaccine, as worldwide MV vaccination prevents an estimated 80 million cases and 4.5 million deaths annually [55,56] with minimal adverse events (on average, less than 10 in 1 million doses) [57]. Additionally, several cancer clinical trials using escalating doses of modified recombinant MV as oncolytic agents have been performed without recording severe adverse events [35,36]. Thus, a novel MV reprogramming system could be directly translated into the clinic, and we are currently working on developing a full MV system to reprogram iPSCs.

## Conclusions

In this study, our goal was to provide a proof of principle that MV can be used as a vector for nuclear reprogramming, and now we have opened the door for the development of increasingly efficient vectors and promising avenues for regenerative medicine.

## Additional files

Additional file 1: Figure S1. Expression of pluripotency-associated markers in measles virus (MV)-derived induced pluripotent stem cell (iPSC)-like clones at passage 15. Three MV- and one lentiviral vector (LV)-derived iPSC clone (#1, #2, #3, and 4LV) were cultured under feeder-free conditions on a Matrigel-based slide and examined for expression of human pluripotent stem cell markers by immunofluorescence at passage 15. Scale bars: 50 μm. Additional file 2: Figure S2. Percentage of cells expressing pluripotency-associated markers in measles virus (MV)-derived induced pluripotent stem cell (iPSC)-like clones. Flow cytometry analyses were performed to determine TRA-1-60-, TRA-1-81-, SSEA1-, SSEA-4-, and OCT4-positive cell populations at passages 6 and 16. Three MV- and one lentiviral vector (LV)-derived iPSC clone (#1, #2, #3, and 4LV) were dissociated and stained with the antibody to the human pluripotent stem cell markers as indicated. Percentages of positive cells are indicated in the top right corner of each panel. Additional file 3: Figure S3. Normal and abnormal karyotypes of the measles virus (MV)-derived induced pluripotent stem cell (iPSC) clones. Most of the cells from clones #1 and #3 have a normal karyotype. Five (clone #1) and six (clone #3) out of 20 cells showed an abnormal karyotype with trisomy 12 or 14, respectively. Clone #2 showed that each metaphase had an isochromosome 20q, resulting in deletion of 20p and duplication of 20q (three total copies of 20q). The result of the karyotype of clone #2 showed that each metaphase also had a single X sex chromosome which could indicate a constitutional or acquired loss of the Y chromosome. Although the predominant low-level abnormality recognized in both mesenchymal stromal cell and iPSC type is trisomy 12, abnormalities involving chromosomes 1, 17, and 20 are also common. Abnormalities of 20q, in particular, have been observed in prolonged culture of iPSCs [44-47]. Additional file 4:
**Movie V1.** Contracting lentiviral vector (LV)-derived induced pluripotent stem cell (iPSC) differentiated cardiomyocyte-like cells. Additional file 5:
**Movie V2.** Contracting MV(OCT4)-derived induced pluripotent stem cell (iPSC) differentiated cardiomyocyte-like cells.

## Abbreviations

ATU: additional transcription unit
DMEM: Dulbecco’s modified Eagle’s medium
EB: embryoid body
FCS: fetal calf serum
FITC: fluorescein isothiocyanate
GFP: green fluorescent protein
iPSC: induced pluripotent stem cell
LV: lentiviral vector
MOI: multiplicity of infection
MV: measles virus
PBS: phosphate-buffered saline
PFA: paraformaldehyde
RT-PCR: reverse transcription-polymerase chain reaction
SCID: severe combined immunodeficiency
SeV: Sendai virus
SPB: saponin permeabilization buffer
TCID_50_: tissue culture infectious dose 50
