# Supplementary figures and images for: Nuclear reprogramming with a non-integrating human RNA virus
Source: Stem Cell Res Ther. 2015 Mar 26;6(1):48. doi: 10.1186/s13287-015-0035-z (PMC4415226; doi:10.1186/s13287-015-0035-z)

Figure S1

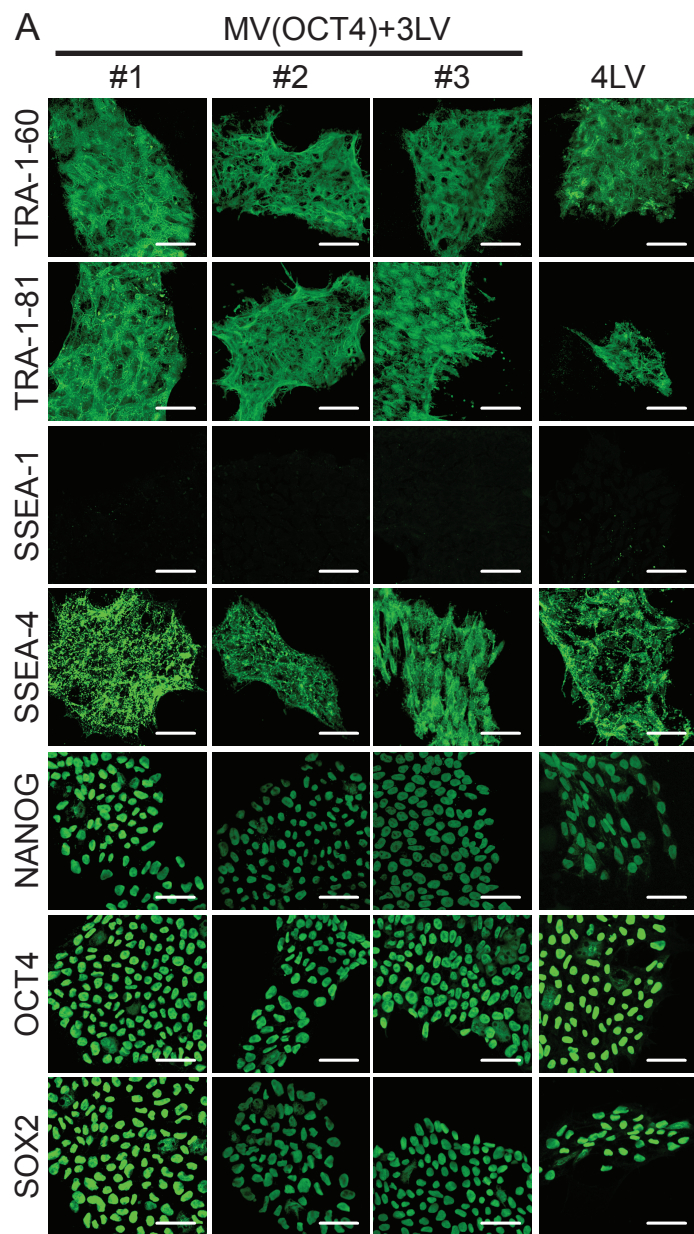

Supplement: Additional file 1: Figure S1. — Expression of pluripotency-associated markers in measles virus (MV)-derived induced pluripotent stem cell (iPSC)-like clones at passage 15. Three MV- and one lentiviral vector (LV)-derived iPSC clone (#1, #2, #3, and 4LV) were cultured under feeder-free conditions on a Matrigel-based slide and examined for expression of human pluripotent stem cell markers by immunofluorescence at passage 15. Scale bars: 50 μm. [file 13287_2015_35_MOESM1_ESM.pdf]

Figure S2

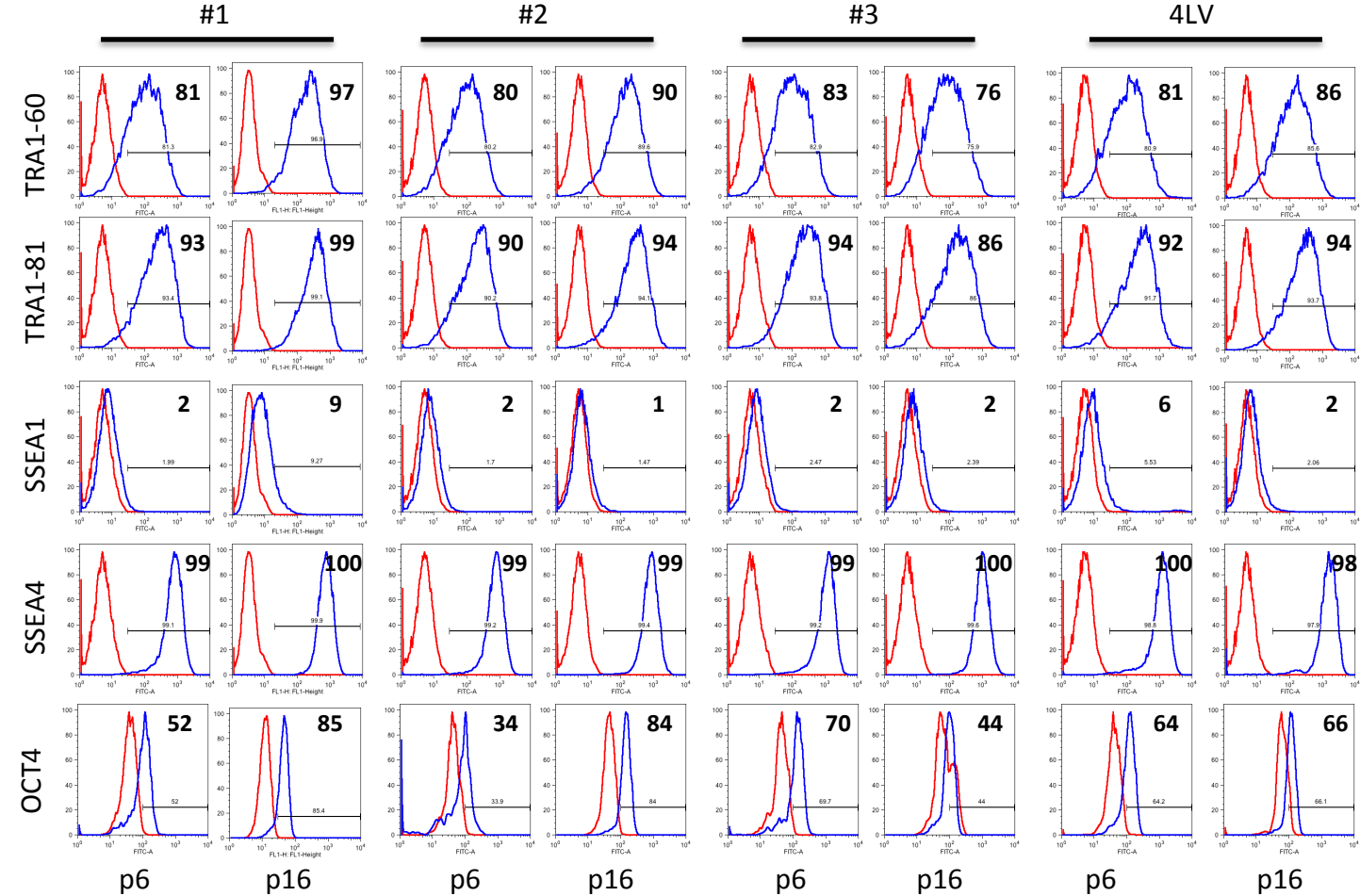

Supplement: Additional file 2: Figure S2. — Percentage of cells expressing pluripotency-associated markers in measles virus (MV)-derived induced pluripotent stem cell (iPSC)-like clones. Flow cytometry analyses were performed to determine TRA-1-60-, TRA-1-81-, SSEA1-, SSEA-4-, and OCT4-positive cell populations at passages 6 and 16. Three MV- and one lentiviral vector (LV)-derived iPSC clone (#1, #2, #3, and 4LV) were dissociated and stained with the antibody to the human pluripotent stem cell markers as indicated. Percentages of positive cells are indicated in the top right corner of each panel. [file 13287_2015_35_MOESM2_ESM.pdf]

Figure S3

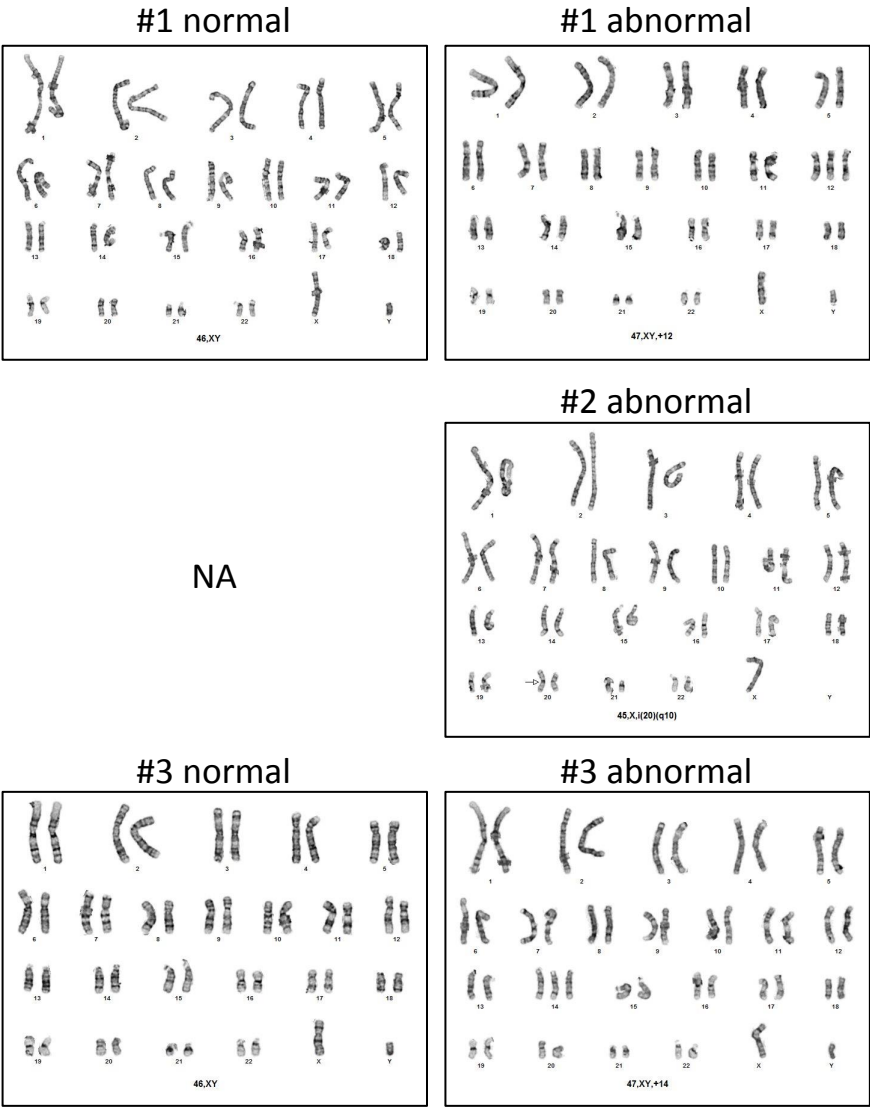

Supplement: Additional file 3: Figure S3. — Normal and abnormal karyotypes of the measles virus (MV)-derived induced pluripotent stem cell (iPSC) clones. Most of the cells from clones #1 and #3 have a normal karyotype. Five (clone #1) and six (clone #3) out of 20 cells showed an abnormal karyotype with trisomy 12 or 14, respectively. Clone #2 showed that each metaphase had an isochromosome 20q, resulting in deletion of 20p and duplication of 20q (three total copies of 20q). The result of the karyotype of clone #2 showed that each metaphase also had a single X sex chromosome which could indicate a constitutional or acquired loss of the Y chromosome. Although the predominant low-level abnormality recognized in both mesenchymal stromal cell and iPSC type is trisomy 12, abnormalities involving chromosomes 1, 17, and 20 are also common. Abnormalities of 20q, in particular, have been observed in prolonged culture of iPSCs [44-47]. [file 13287_2015_35_MOESM3_ESM.pdf]
